# Demonstrating the relationship of ultrasonographic parameters with disease activity and pain in lateral epicondylitis

**DOI:** 10.1097/MD.0000000000035499

**Published:** 2023-10-06

**Authors:** Emre Bal, Onur Cetin

**Affiliations:** a Fatih Sultan Mehmet Education and Training Hospital Orthopedics and Traumatology Department, Istanbul, Turkey; b Medipol University, Camlica Hospital, Orthopedics and Traumatology Department, Istanbul, Turkey.

**Keywords:** lateral epicondylitis, musculoskeletal ultrasound, pain, tennis elbow

## Abstract

To evaluate the relationship of ultrasonographic evaluation parameters with pain, muscle strength and disease severity in lateral epicondylitis (LE). 64 people were included in present retrospective, cross-sectional study. Activity and rest pain was questioned with Visual Analog Scale (VAS). Also, Patient Rated Tennis Elbow Evaluation (PRTEE) and the maximum grip strength were evaluated. Hypoechoic region, neovascularity, cortical irregularity, enthesopathy and peritendinous fluid or bursitis were evaluated by ultrasonography. 48 of the patients were female and 16 were male. Mean age was 48.53 ± 6.12, body mass index was 27.70 ± 4.75. 55 (85.9%) hypoechoic region, 31 (48.4%) neovascularity, 21 (32.8%) cortical irregularity, 19 (29,7%) enthesopathy, and 18 (28.1%) peritendinous fluid or bursitis were detected by ultrasonography. When the ultrasonographic findings and clinical findings of the patients were compared, no significant difference was found between the hypoechoic region, cortical irregularity, enthesopathy and clinical findings (*P* > .05), while the extension grip strength was found to be significantly lower in patients with neovascularity (*P* = .045). In addition, patients with peritendinous fluid or bursitis, were found to be significantly lower in both flexion (*P* = .033) and extension (*P* = .023) grip strength, while PRTEE function (*P* = .021) subgroup and total (*P* = .038) scores were significantly higher. Hypoechoic region, cortical irregularities and enthesopathy were not evaluated to be associated with disease severity, pain and muscle strength. Neovascularity was found to be associated only with extension grip strength. Peritendinous fluid or bursitis was found to be associated with both flexion and extension grip strength and disease activity, but not associated with pain.

## 1. Introduction

Lateral epicondylitis (LE) is the most common cause of discomfort in the lateral region of the elbow, characterized by pain in that area.^[1,2]^ It affects approximately 1.3% of the general population.^[3]^ LE is caused by pathological alterations in the tendons of the extensor carpi radialis brevis, extensor carpi ulnaris, extensor digitorum, and extensor digiti minimi muscles, which comprise the same extensor origin. Currently, there is still not a definitive method for diagnosing LE. However, the Cozen, Mill, and Maudsley tests are frequently used provocation tests that are regarded as positive if they duplicate lateral elbow discomfort in clinical practice and research.^[4]^ As a result of the lack of a gold standard diagnostic technique, variable diagnostic tools are needed. Musculoskeletal ultrasound (MUS) is a reliable, accessible, noninvasive, and cost-effective option for diagnosis.^[5,6]^ Additionally, MUS offers the chance for a dynamic examination, giving it an advantage over MRI and CT.^[7]^ When it comes to diagnosing epicondylitis, sonography is sensitive but not specific as MRI.^[8]^ Gray-scale ultrasonography (USG) can be used to identify LE with intermediate sensitivity and high specificity.^[1]^ MUS can reveal bone abnormalities, calcifications, enthesopathy, localized hypoechoic regions, peritendinous fluid, linear intrasubstance tears, and thickening of the tendon in tendinosis of the common extensor tendon.^[9]^

Previous studies have provided evidence for the diagnostic utility of MUS in LE. However, there is a lack of understanding regarding the relationship between ultrasonographic evaluations and the severity of the condition. The objective of our study was to examine the correlation between pain, muscle strength, and disease severity in LE using ultrasonographic evaluation measures.

## 2. Methods

Our study was carried out in a single-center at Fatih Sultan Mehmet Training and Research Hospital, Department of Orthopedics and Traumatology between January 2021 and February 2023. Prior to the study, participants provided consent. Ethical approval was obtained from the Agri İbrahim Cecen University Ethics Committee, adhered to the principles of the Declaration of Helsinki.

In this study, we enrolled 64 patients who were over the age of 18 and sought treatment at our clinic for elbow pain, diagnosed with LE after physical examination, no other pathology detected on direct radiographs. Additionally, these patients had been experiencing their symptoms for more than 6 months despite previous conservative treatments. We collected demographic information and medical history from all participants.

In the physical examination, palpation of the LE and Mills, Maudsley and Cozen tests were performed. The patients were also asked to rate their level of activity and rest pain using the Visual Analogue Scale (between 0 and 10 points). Jamar hydraulic hand dynamometer, considered as the gold standard by the American Association of Hand Therapists, was utilized to assess the rough grip strength.^[10]^ For painless grip strength evaluation, patients were instructed to squeeze the dynamometer until they felt discomfort.

The evaluation of maximum grip strength was conducted to assess the strength reached by patients beyond their pain threshold. The first test was performed with patients seated in a chair with their arms supported, shoulder in adduction and neutral position, elbow flexed at 90°, forearm in a neutral position, and wrist in a position of 0° to 30° extension and 0° to 15° ulnar deviation. The second test was performed with patients in a standing position, shoulder adducted and elbow extended. A 30-second rest period was given between each measurement.

In this study, the ultrasonographic examination was performed with the Esaote Mylab 60 device using a linear probe with a frequency of 6 to 18 MHz. During the examination, the patients were positioned with their shoulders adducted, elbows flexed at 90 degrees, and wrists pronated. The USG probe was placed longitudinally on the radial surface of the elbow to measure the thickness of the common extensor tendon.

Patient Rated Tennis Elbow Evaluation (PRTEE) formerly known as the Patient-Rated Forearm Questionnaire was developed by Overand et al This evaluation form is designed specifically for patients with LE. It consists of 2 sections that assess pain and function. The function subheading includes specific activities and general activities. Each subsection takes a value between 0 and 100. For the total score, the pain total score and the average of the specific and general activity scores are added. As a result, a value between 0 and 100 is obtained.^[11]^

### 2.1. Statistical analysis

Statistical analysis of all the study data was performed using IBM SPSS Statistics for Windows, Version 25.0. Descriptive data were expressed in mean ± standard deviation or number and frequency. The distribution of variables was checked with the Kolmogorov–Smirnov test. To compare between 2 groups, an independent sample *t* test or Mann–Whitney *U* test was performed for quantitative variables. The value *P* < .05 was accepted to indicate statistical significance.

## 3. Results

64 individuals who met the inclusion criteria were included in this retrospective study. 48 of the patients were female and 16 were male. Mean age was 48.53 ± 6.12, body mass index was 27.70 ± 4.75, and duration of complaint was 20.37 ± 22.19 months. While the dominant hand was the right hand in 62 (96.8%) patients and the left side in 2 (3.1%) patients, 41 patients (64.1%) reported complaints on the right elbow, and 23 patients (35.9%) on the left elbow. While 29 (45.3%) of the patients included in the study were actively working, 35 (54.7%) were not working.

While the mean Visual Analog Scale (VAS) values of the patients were 3.28 ± 2.30 at rest, it was 7.43 ± 1.62 during activity. In the PRTEE evaluation, the pain sub-values were 29.54 ± 5.37, the function sub-evaluation was 30.61 ± 7.21, and the total values were 60.16 ± 11.22. When the painless values of the patients’ handgrip strength were examined, the mean values were 14.37 ± 7.19 in the elbow flexion position and 12.54 ± 7.15 in the extension position. While the tendon thickness measurements of the patients were 4.61 ± 0.60 on the sick side, it was 4.55 ± 0.60 on the healthy side, and there was no statistically significant difference between the 2 sides (*P* = .609).

MUS findings of the patients with LE were examined. 55 (85.9%) hypoechoic region, 31 (48.4%) neovascularity, 21 (32.8%) cortical irregularity, 19 (29.7%) enthesopathy, and 18 (28.1%) peritendinous fluid or bursitis were detected. MUS findings and clinical findings of the patients were compared and no significant difference was found between the hypoechoic region, cortical irregularity, enthesopathy and clinical findings (*P* > .05), while the extension grip strength was found to be significantly lower in patients with neovascularity (*P* = .045). In addition, in patients with peritendinous fluid or bursitis, was found to be significantly lower in both flexion (*P* = .033) and extension (*P* = .023) grip strength, while the PRTEE function subgroup (*P* = .021) and total scores (*P* = .038) were significantly higher. The results are shown in Table 1.

**Table 1:** Comparison of ultrasonographic parameters of the participants with muscle strength and Patient Rated Tennis Elbow Evaluation (PRTEE) data.

	VAS rest	VAS Activity	Jamar Flex	Jamar Ext	PRTEE pain	PRTEE function	PRTEE total
Hypoechoic regions Yes (n = 55) No (n = 9)	3.32 ± .2.24 3.00 ± 2.78	6.88 ± 1.26 7.52 ± 1.66	14.07 ± 6.41 15.44 ± 11.30	6.63 ± 12.18 6.61 ± 12.77	29.72 ± 5.19 28.44 ± 6.59	30.60 ± 7.28 30.72 ± 7.21	60.32 ± 11.03 59.16 ± 12.97
*P* value	0.519	0.298	0.459	0.809	0.656	0.931	0.721
Neovascularity Yes (n = 31) No (n = 33)	3.09 ± 2.24 3.45 ± 2.38	7.38 ± 1.43 7.48 ± 1.80	13.35 ± 6.99 15.33 ± 7.35	10.70 ± 6.58 14.27 ± 7.31	29.77 ± 4.83 29.33 ± 5.89	31.96 ± 6.75 29.34 ± 7.50	61.74 ± 10.02 58.68 ± 12.20
*P* value	0.538	0.812	0.274	0.045*	0.746	0.147	0.279
Cortical irregularities Yes (n = 21) No (n = 43)	3.76 ± 2.14 3.04 ± 2.36	7.42 ± 1.46 7.44 ± 1.66	14.42 ± 6.42 14.34 ± 7.61	10.76 ± 6.00 13.41 ± 7.54	30.76 ± 4.97 28.95 ± 5.51	30.30 ± 6.71 30.76 ± 7.52	61.07 ± 10.06 59.72 ± 11.83
*P* value	0.247	0.976	0.965	0.134	0.195	0.807	0.665
Enthesopathy Yes (n = 19) No (n = 45)	3.36 ± 2.29 3.24 ± 2.33	7.47 ± 1.50 7.42 ± 1.68	13.57 ± 6.70 14.71 ± 7.44	12.26 ± 7.19 12.66 ± 7.38	30.05 ± 5.60 29.33 ± 5.32	31.81 ± 7.68 30.11 ± 7.04	61.86 ± 11.16 59.44 ± 11.29
*P* value	0.845	0.905	0.569	0.839	0.628	0.412	0.434
Peritendinous fluid or bursit Yes (n = 18) No (n = 46)	3.44 ± 2.20 3.21 ± 2.36	7.66 ± 1.78 7.34 ± 1.56	11.33 ± 5.85 15.56 ± 7.37	9.38 ± 6.41 13.78 ± 7.09	30.66 ± 4.63 29.10 ± 5.61	33.91 ± 6.67 29.32 ± 7.07	64.58 ± 9.78 58.43 ± 11.63
*P* value	0.719	0.484	0.033*	0.023*	0.263	0.021*	0.038*

Neovascularity and extension grip strength were found to be significantly lower (*P* = .045). While both flexion (*P* = .033) and extension (*P* = .023) grip strength was found to be significantly lower in patients with peritendinous fluid or bursitis, the PRTEE function subgroup (*P* = .021) and total score (*P* = .08) were significantly higher.

Ext = extension, Flex = flexion, PRTEE = Patient Rated Tennis Elbow Evaluation, VAS = Visual Analog Scale.

## 4. Discussion

In this study, we aimed to investigate the relationship between MUS parameters and clinical parameters in LE. As a result of our study, hypoechoic region, cortical irregularities and enthesopathy were evaluated not to be associated with disease severity, pain and muscle strength. Neovascularity was found to be associated only with muscle strength of wrist extension. Peritendinous fluid or bursitis was found to be associated with both grip strength of wrist flexion and extension and disease activity, but not linked with pain.

In a meta-analysis, female gender reported as a risk factor for LE with an Odds ratio of 0.77.^[12]^ Likewise, in our study female gender was reported as a dominant gender.

Maffulli et al categorized sonographic findings in LE into 2 groups: tendinous disease and extratendinous pathology, which included enthesopathy, tendinitis, peritendinitis, and mixed lesions.^[13]^ Additionally, bone alterations in the lateral epicondyle were found to be mildly associated with chronic LE. However, cortical abnormalities and neovascularity calcifications exhibited high specificity for persistent lateral epicondylalgia.

Various techniques such as real-time sonoelastography, color Doppler USG, and sonographic probe-induced tenderness have not been widely researched for LE diagnosis.^[1]^ In a study, tendinosis, hypoechogenicity, hyperechogenicity, edema, calcification, enthesopathy, and bursitis signs were observed in 75% of the patients with LE during MUS evaluation.^[14]^ The sensitivity and specificity of grayscale USG in diagnosing LE have varied in previous reports, ranging from 36% to 100% and 72% to 88%, respectively.^[3,8,15]^ And in patients with tennis elbow, the sensitivity and specificity of Doppler USG are 95% and 88%, respectively.^[3,16]^ Levin et al reported low specificity and high sensitivity for USG of the common extensor tendon in their investigation of LE.^[15]^ Additionally, they discovered statistical significance for the symptoms, tendon thickening, focal hypoechoic areas, uneven bone at the attachment site, intratendinous calcification, and widespread heterogeneity.^[15]^

It is appropriate to use high-frequency linear transducers when examining structures around the elbow with ultrasonography. MUS findings in tendinosis of the common extensor tendon can include tendon thickening, focal hypoechoic regions, peritendinous fluid, linear intrasubstance tears, bone irregularities, calcifications, enthesopathy, and diffuse tendon heterogeneity.^[15]^ Hypoechoic tendon thickening in epicondylitis may be complicated by partial tears, fissures, calcifications, or synovitis. Cortical irregularities and spur formation can be observed in the epicondyle.^[9]^ Tendon thickness can be diagnosed if the tendon on the sick side is increased by more than 30% compared to the healthy side.^[14]^ Lee et al demonstrated that the common extensor tendon was significantly thicker in patients with LE than in control subjects, with a tendon thickness >4.2 mm and an area larger than or equal to 32 mm^2^ were highly predictive of LE.^[17]^

In a study, comparing the ultrasonographic changes on 60 patients with LE and 264 participants with no symptoms demonstrated the tendon thickness and color Doppler activity on LE patients. They concluded that the tendon thickness cannot be used as a stand-alone diagnostic tool, however color Doppler activity is a marker of the ongoing tendinopathy and supports the diagnosis of LE.^[2]^ In harmony with this study, Zeisig et al found a positive color Doppler activity in 95% of LE patients and 9% of control group.^[18]^ Another study evaluating color Doppler activity demonstrated a pathological vascularity in 33 of 35 patients (94%).^[19]^

Struijs et al discovered sonographic abnormalities in 75% of the patients, including hypoechoic and hyperechoic regions, swelling, calcification, bursitis, enthesopathy, and tendinosis. The positive predictive value of sonography ranged from 0.78 and 0.82, and the negative predictive value ranged from 0.23 and 0.71. When the predictive value was examined among the subgroups, it was not found to be associated with treatment success and pain.^[14]^ Lee et al found that hypoechoic regions (35.3%), peritendinous fluid (3.9%), and cortical irregularities (17.6%) had low sensitivity for LE.^[17]^ Although focal hypoechogenicity was moderately sensitive and highly specific for LE, it was not found to be associated with pain, muscle strength, and disease severity.^[1]^ Our study results align with these findings, as we also found no relationship between hypoechoic regions and disease severity, pain, or muscle strength. Bone changes in the lateral epicondyle were moderately susceptible to chronic lateral epicondylalgia. Although cortical irregularities have been suggested as features of the chronic stage of musculoskeletal disease, they are less frequently detected in lateral epicondylalgia elbows compared to focal hypoechogenicity.^[20]^

Furthermore, the relationship between calcifications, which is considered to be the main feature of degenerative tendon changes, and lateral epicondylalgia was found inconsistent. This may indicate that pathological changes in elbows with LE are more traumatic than degenerative.^[1]^ Cortical irregularities and hypoechoic regions, which have limited diagnostic value, were not associated with pain, functionality, or wrist flexion and extension muscle strength in our study. Bone spur could not be associated with outcomes such as pain, disability, PRTEE, and disease duration. However, pain and functionality assessments in cases of peritendinous fluid or bursitis were related to wrist flexion and extension muscle strength. Additionally, color Doppler detected peritendinous hyperemia or neovascularity in the intratendinous region. The absence of neovascularity and grayscale changes on MUS examination should prompt consideration of other causes of lateral elbow pain.^[2]^ Conversely, although healthy elbows usually do not have neovascularity, there are studies with inconsistent results in the diagnostic ability to confirm the presence of lateral epicondylalgia in painful elbow.^[3,21]^

Similarly, Yuill et al identified neovascularity, calcifications, and cortical irregularities as highly specific but weakly sensitive indicators of chronic LE. There is little clarity about the role of these findings in the diagnosis of LE.^[21]^ In our study, neovascularity, despite its diagnostic value for LE, was not associated with pain, functionality, or wrist flexion muscle strength. Similarly, du Toit et al found that common extensor tendon neovascularity and clinical severity measures did not correlate with neovascularity scores.^[3]^ Krogh et al also did not find a relationship between Doppler activity and outcomes such as pain, disability, PRTEE, or disease duration.^[2]^ In contrast, in our study, it was found to be associated with neovascularity and wrist extension muscle strength.

Our study has several limitations. The diagnosis of LE was made clinically and the differences in body structure were not taken into account in the ultrasonographic evaluation. The results of our study are limited due to the small number of patients participating in the study. We recommend planning future studies with a larger sample size.

One of the strengths of our study is that patients’ muscle strength was evaluated with the Jamar hand dynamometer, which is considered the gold standard. Disease activity was assessed with a large-scale questionnaire that included both pain and function and reflects disease exposure in detail. The use of both gray and color Doppler is one of the strengths of our study.

## 5. Conclusion

Hypoechoic regions, cortical irregularities and enthesopathy were not evaluated to be associated with disease severity, pain and muscle strength. Neovascularity was found to be associated only with muscle strength of wrist extension. Peritendinous fluid or bursitis was found to be associated with both muscle strength of wrist flexion and extension and disease activity, but not associated with pain. Demonstrating the relationship of ultrasonographic parameters used in the evaluation of LE with pain, disease severity and muscle strength may provide an idea for the use of ultrasonographic evaluation in the effectiveness of LE diagnosis and treatments.

## Acknowledgments

We thank for the Radiology Department of the Fatih Sultan Mehmet Education and Research Hospital for their ultrasonographic evaluation on patients.

## Author contributions

**Data curation:** Emre Bal.

**Formal analysis:** Onur Cetin.

**Investigation:** Emre Bal.

**Methodology:** Onur Cetin.

**Project administration:** Emre Bal.

**Resources:** Emre Bal.

**Visualization:** Emre Bal.

**Writing – original draft:** Emre Bal.

**Writing – review & editing:** Onur Cetin.
